# Glutamine deprivation induces ferroptosis in pancreatic cancer cells: Glutamine deprivation induces pancreatic cancer ferroptosis

**DOI:** 10.3724/abbs.2023029

**Published:** 2023-03-20

**Authors:** Zhiwen Xiao, Shengming Deng, He Liu, Ruijie Wang, Yu Liu, Zhengjie Dai, Wenchao Gu, Quanxing Ni, Xianjun Yu, Chen Liu, Guopei Luo

**Affiliations:** 1 Department of Pancreatic Surgery Fudan University Shanghai Cancer Center Shanghai 200032 China; 2 Department of Oncology Shanghai Medical College Fudan University; Shanghai Pancreatic Cancer Institute Shanghai 200032 China; 3 Pancreatic Cancer Institute Fudan University Shanghai 200032 China; 4 Department of Diagnostic Radiology and Nuclear Medicine Gunma University Graduate School of Medicine Maebashi Gunma 371-8511 Japan

**Keywords:** ferroptosis, glutamine, glutathione, pancreatic cancer, reactive oxygen species

## Abstract

Ferroptosis is a type of programmed cell death closely related to amino acid metabolism. Pancreatic cancer cells have a strong dependence on glutamine, which serves as a carbon and nitrogen substrate to sustain rapid growth. Glutamine also aids in self-protection mechanisms. However, the effect of glutamine on ferroptosis in pancreatic cancer remains largely unknown. Here, we aim to explore the association between ferroptosis and glutamine deprivation in pancreatic cancer. The growth of pancreatic cancer cells in culture media with or without glutamine is evaluated using Cell Counting Kit-8. Reactive oxygen species (ROS) are measured by 2′,7′-dichlorodihydrofluorescein diacetate staining. Ferroptosis is assessed by BODIPY-C11 dye using confocal microscopy and flow cytometry. Amino acid concentrations are measured using ultrahigh-performance liquid chromatography-tandem mass spectrometry. Isotope-labelled metabolic flux analysis is performed to track the metabolic flow of glutamine. Additionally, RNA sequencing is performed to analyse the genetic alterations. Glutamine deprivation inhibits pancreatic cancer growth and induces ferroptosis both *in vitro* and *in vivo*. Additionally, glutamine decreases ROS formation via glutathione production in pancreatic cancer cells. Interestingly, glutamine inhibitors (diazooxonorleucine and azaserine) promotes ROS formation and ferroptosis in pancreatic cancer cells. Furthermore, ferrostatin, a ferroptosis inhibitor, rescues ferroptosis in pancreatic cancer cells. Glutamine deprivation leads to changes in molecular pathways, including cytokine-cytokine receptor interaction pathways ( *CCL5*, *CCR4*, *LTA*, *CXCR4*, *IL-6R*, and *IL-7R*). Thus, exogenous glutamine is required for the detoxification of ROS in pancreatic cancer cells, thereby preventing ferroptosis.

## Introduction

Pancreatic ductal adenocarcinoma (PDAC) is one of the most lethal neoplasms, with an average five-year survival rate of less than 10% [1]. Unfortunately, most patients present with unresectable tumors at the time of diagnosis. Only 20% of patients have the opportunity for curative resection [1]. Although surgical interventions have made promising improvements in recent decades, long-term outcomes rely heavily on adjuvant treatments, even in patients with resectable pancreatic cancer [2]. Among the adjuvant treatments, chemotherapy regimens, including FOLFIRINOX and gemcitabine, have shown encouraging results. However, their therapeutic efficacies and side effects remain unsatisfactory [2]. Accurate termination of cancer cells without affecting normal tissues is a difficult goal. Therapies that can selectively target cancer cells are considered promising methods for treating cancers [3]. Since pancreatic cancer cells rely on reprogrammed metabolic pathways to survive and progress, blocking rewired metabolic networks might be an appealing therapeutic approach.

Ferroptosis is a type of regulated cell death dependent on iron. It is genetically and biochemically different from other forms of programmed cell death, such as apoptosis, necroptosis, and autophagy [4]. Ferroptosis is characterized by the accumulation of lipid peroxides. It is initiated by the failure of glutathione-dependent antioxidant defenses, leading to unchecked lipid peroxidation, catastrophic reactive oxygen species (ROS) accumulation, and eventual cell death [5]. Ferroptosis was discovered when a small-molecule agent, erastin, was identified during screening [6]. Erastin, coined from “Eradicator of RAS- and ST-expressing cells,” is selectively detrimental to tumor cells harboring oncogenic *RAS* mutations [6]. Since *KRAS* mutations are present in over 90% of pancreatic cancers, they may serve as an ideal model for studying ferroptosis. Moreover, the translational significance is greater considering that *KRAS* is largely an undruggable target. Interestingly, tumors harboring *KRAS* mutations have proven to be vulnerable to ferroptosis [7].

Glutamine is the most abundant free amino acid in the human body [8]. Despite being a nonessential amino acid, glutamine, which has been viewed as a nitrogen trap, is an essential source of carbon and nitrogen during the development of cancer [ 9, 10] . Cancer cells consume endogenous and/or exogenous glutamine to support their growth in a context-dependent manner [9]. Compared with other cancer types, pancreatic cancer cells have a stronger dependence on glutamine, as it serves as a source of carbon and nitrogen to fulfil their rapid growth, self-protection mechanisms, invasion, and metastasis [ 11, 12] . For example, glutamine blockade not only suppresses the metabolic programs of pancreatic cancer cells but also enhances their antitumour immune response by modulating CD8 ^+^ tumor-infiltrating lymphocytes toward a highly proliferative, activated, and long-lived phenotype [12]. Therefore, the pathways involved in glutamine metabolism could be exploited for therapeutic purposes in pancreatic cancer.

Although glutamate is a proven ferroptosis inhibitor, its effect on ferroptosis in pancreatic cancer has not been extensively studied [4]. In the present, we examined the possible association between ferroptosis and glutamine deprivation. The potential mechanisms involved in ferroptosis induction upon glutamine deprivation were also investigated.

## Materials and Methods

### Cells and culture conditions

BxPC-3, SU.8686, SW1990, CAPAN-1, CAPAN-2, CFPAC-1, MiaPaCa-2, and Panc-1 human pancreatic cancer cell lines were obtained from the American Type Culture Collection (Manassas, USA). All cells were maintained in Dulbecco’s modified Eagle’s medium (DMEM) or Roswell Park Memorial Institute (RPMI)-1640 supplemented with 4.5 g/L glucose, 10% fetal bovine serum (FBS), and 1% penicillin/streptomycin (Thermo Fisher Scientific, Waltham, USA ) in a humidified incubator at 37°C with 5% CO _2_. In this study, DMEM without glutamine (Gibco, Carlsbad, USA; Cat. #2120455) was supplemented with sodium pyruvate (Gibco; Cat. #2085655). Additionally, DMEM containing different concentrations of glutamine was used. Only mycoplasma-negative cells were used in this study.

### Animal models

Six-week-old female athymic BALB/c nude mice were obtained from the Shanghai Jihui Laboratory Animal Co., Ltd. (Shanghai, China) and maintained in specific pathogen-free facilities according to the approved experimental protocols. The animals were maintained in a temperature-controlled, air-conditioned animal house (20±4°C, 55%±10% humidity). The mice were housed in individual cages in an open-cage system and received food and water *ad libitum*. The right flanks of the mice were injected subcutaneously with 1×10 ^6^ SW1990 (seven mice in each group) or 1×10 ^6^ BxPC-3 (six mice in each group) pancreatic cancer cells. Mice were randomly assigned to either the diazooxonorleucine (Don) group (750 μg/kg) or the control vehicle group (same volume of saline). The drugs were intraperitoneally administered after inoculation biweekly. The tumor sizes of the xenografts were measured twice a week in length and width using callipers. Tumor volumes were calculated using the following formula: Tumor volume=(long axis) (short axis) ^2^ π/6. The mice were euthanized using 1% pentobarbital sodium in physiological saline 30 days after tumor inoculation. The tumors were surgically dissected and weighed. The animal study protocol was reviewed and approved by the Ethical Committee of the Fudan University Shanghai Cancer Center (No. FUSCC-IACUC-S20210186).

### Cell counting kit-8 (CCK-8) assay

Cell viability was evaluated every 24 h using a Cell Counting Kit-8 (DOJINDO, Kumamoto, Japan) according to the manufacturer’s instructions. Briefly, 3000 cells were seeded per well in 96-well plates and cultured for the indicated days. To avoid the influence of the drug on the absorbance, the original medium was removed, and 100 μL of medium containing the CCK-8 reagent was added to the cells and further incubated at 37°C for 2‒3 h. Absorbance was measured at 450 nm with a Synergy H4 Hybrid Microplate Reader (BioTek, Winooski, USA). A control was provided for each cell line at the indicated time points.

### Reverse transcription-quantitative PCR (RT-qPCR)

Pancreatic cancer cells SW1990, BxPC-3, CaPan-2 and SU.8686 were cultured in media with different concentrations of glutamine (0 and 2 mM). RT-qPCR was performed to evaluate the relative expression levels of *IL6R* and *CXCR4*. Total RNA was isolated from cell lines using the EZ-press RNA Purification kit (EZBioscience, Shanghai, China), followed by cDNA synthesis using a Color Reverse Transcription kit (EZBioscience). The reverse transcription of RNA into cDNA was performed according to the manufacturer’s protocol. The product were amplified using SYBR-Green Premix DimerEraser (Takara, Dalian, China) on theLightCycler480 II system (Roche, Basel, Switzerland). The relative expression was determined using the 2 ^‒ΔΔCq^ method. qPCR was performed under the following conditions: initial denaturation at 95°C for 3 min followed by 40 cycles of 95°C for 30 s, 60°C for 30 s and 72°C for 30 s, with a final extension at 72°C for 1 min. *β-Actin* was selected as an internal reference, to compare gene expression in different samples. The following primers were used: *IL6R* (forward: 5′-GGCTCTGAAGGAAGGCAAGA-3′, reverse: 5′-CTGGCATCTGGTCGGTTGT-3′); *CXCR4* (forward: 5′-CAGTGAGGCAGATGACAG-3′, reverse: 5′-ACAATACCAGGCAGGATAAG-3′); and *β-actin* (forward: 5′-CGTGCGTGACATTAAGGAAGAGT-3′, reverse: 5′-GGAAGGAAGGCTGGAAGAGT-3′).

### ROS evaluation by dichlorodihydrofluorescein diacetate (DCFH-DA) staining

Pancreatic cancer cells were incubated for 72 h in 60 mm ^2^ culture dishes with glutamine-free medium, medium containing 2 mM glutamine, or medium containing 2 mM glutamine supplemented with 50 μM diazooxonorleucine (Cat. #HY-108357; MedChemExpress, Shanghai, China) or 10 μM azaserine (Aza; Cat. #HY-B0919; MedChemExpress). Then, the cells were incubated with 10 μM 2’,7’-DCFH-DA (Cat. #D6883; Sigma-Aldrich, St Louis, USA) in serum-free DMEM at 37°C for 30 min. Cells treated with 100 μM H _2_O _2_ were used as positive controls. Thereafter, cells were treated with glutamine-free medium containing 2 μM ferrostatin-1 (Cat. #HY-100579; MedChemExpress), 5 mM N-acetylcysteine (NAC, Cat. #HY-B0215; MedChemExpress), 50 μM Z-VAD-FMK (Cat. #HY-16658B; MedChemExpress), and 150 μM necrostatin-1 (Cat. #HY-15760; MedChemExpress) to counteract cell death. The cells were then washed twice, resuspended, and filtered into single-cell suspensions before analysis. The fluorescence intensity of DCFH, formed by the reaction of DCFH-DA dyes with ROS, was measured at excitation and emission wavelengths of 488 and 530 nm, respectively, with a FC500 MPL flow cytometer (Beckman Coulter, Pasadena, USA). A minimum of 10,000 cells were analyzed per condition [13]. Proportion analysis and histogram generation were performed using FlowJo software (version 10.6; FlowJo, Ashland, USA).

### BODIPY-C11 analysis by confocal microscopy and flow cytometry

For confocal imaging, 10,000 cells were seeded per well in a 24-well plate containing glass slides that were pretreated with polylysine. Cells were treated with glutamine-free medium, medium containing 2 mM glutamine, or medium containing 2 mM glutamine supplemented with 50 μM Don or 10 μM Aza for 72 h. Cells treated with 10 μM RSL3 (Cat. #S8155; Selleck, Housto, USA) or 20 μM erastin (Cat. #HY-15763; MedChemExpress) were used as positive controls. The samples were incubated with 1 μM BODIPY-C11 dye (Cat. #D3861; Thermo Fisher Scientific) for 30 min. After being washed with FBS-free DMEM, the slides were mounted using DAPI Fluoromount-G (Southern Biotech, Birmingham, USA). The cells were imaged at 63× oil magnification using a Leica SP5 II confocal laser scanning microscope (Leica, Wetzlar, Germany). Image analysis was performed using Image-Pro Plus software (Media Cybernetics, Rockville, USA). At least seven randomly selected fields were analyzed per sample.

Cells were seeded in 6-well plates for flow cytometry analysis. At the indicated time points, cells were removed along with the medium, washed with prewarmed PBS, and incubated with 5 μM BODIPY-C11 dye diluted in FBS-free DMEM. The cells were washed with DMEM and filtered into single-cell suspensions prior to flow cytometry analysis. Flow cytometry was performed on an FC500 MPL flow cytometer (Beckman Coulter). Oxidized BODIPY-C11 (green emission at 510 nm) was detected. A minimum of 10,000 cells were analyzed for each condition. Data analysis was performed using FlowJo software (version 10.6). The fold increase in BODIPY-C11 ^+^ cells was calculated by dividing the percentage of ferroptotic cells in cells cultured in glutamine-free media.

### RNA sequencing

RNA sequencing was performed as previously reported with some modifications [14]. Briefly, total RNA was extracted from the cell samples using Trizol reagent (Invitrogen, Carlsbad, USA). Sequencing was conducted on a BGIseq500 platform (BGI-Shenzhen, Shenzhen, China) to obtain single-end 50 base-pair reads. Quality control of the sequencing data was performed using SOAPnuke (v1.5.2). PossionDis with a false discovery rate (FDR)≤0.001 and |Log2Ratio|≥1 were used to assess differential gene expression. Gene set enrichment analysis (GSEA) ( http://www.gsea-msigdb.org/) and Kyoto Encyclopedia of Genes and Genomes (KEGG) ( https://www.kegg.jp/) enrichment analysis of the differentially expressed genes were performed.

### Ultrahigh-performance liquid chromatography (UHPLC)-MS/MS

UHPLC-MS/MS was performed as previously reported, with some modifications [15]. For metabolite collection, 1000 μL of the extraction solvent (precooled at ‒20°C; acetonitrile:methanol:water=2:2:1) was added to pancreatic cancer cells, and the contents were vortexed for 30 s, homogenized at 45 Hz for 4 min, and sonicated for 5 min in an ice-water bath. The UHPLC assay was performed using an Agilent 1290 Infinity II series UHPLC System (Agilent Technologies, Santa Clara, USA) and a Waters ACQUITY UHPLC BEH Amide column (100 mm ×2.1 mm,1.7 μm; Waters, Milford, USA).

### Isotope labelled metabolic flux analysis

SU.8686 cells were cultured for 12 or 24 h in media containing L-glutamine- ^15^N _2_ (Cat. #490032; Sigma-Aldrich) or L-Glutamine- ^13^C _5_ (Cat. #605166; Sigma-Aldrich). Cells cultured in unlabelled glutamine were used as controls. A total of 1×10 ^7^ cells were collected for analysis. For sample preparation, 1000 μL of extraction solution (acetonitrile:methanol:water=2:2:1) containing an isotopically labelled internal standard mixture was added to the sample. LC-MS/MS analyses were performed using a UHPLC System (1290; Agilent Technologies) with a UPLC HSS T3 column (2.1 mm× 100 mm,1.8 μm) coupled to a Q Exactive mass spectrometer (Thermo Fisher Scientific). The raw data were converted to the mzXML format using ProteoWizard and processed with an in-house program that was developed using R and based on XCMS for peak detection, extraction, alignment, and integration [16]. M+ (M0, M1 to Mn) represents the number of C or N atoms in the metabolites labelled by the isotopes. The fractional abundances of the mass isotopomers defined by M0, M1, and Mn were calculated.

### Statistical analysis

Data are expressed as the mean±SEM unless otherwise indicated. Statistical analyses were conducted using SPSS software (version 19.0; IBM Corp., Armonk, USA) and Prism statistical software (version 8; GraphPad Software, Inc., La Jolla, USA). Unpaired two-tailed Student’s *t* test was used to compare the data between the two groups. For multiple comparisons, ANOVA combined with the Bonferroni correction was used. Significance was defined as a *P* value <0.05, unless otherwise stated.

## Results

### Glutamine deprivation inhibits pancreatic cancer growth

**Figure 1:**
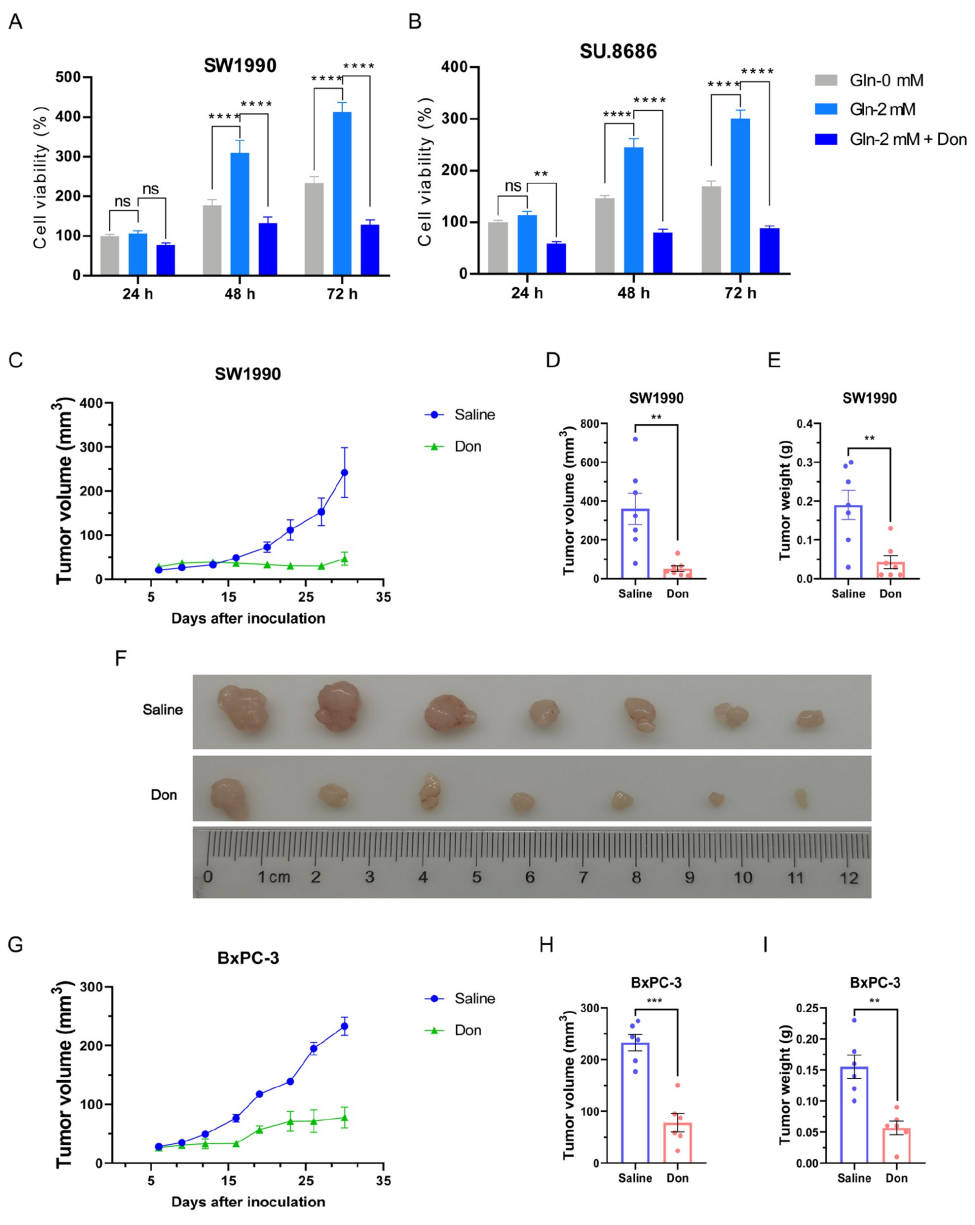
Figure 1 Glutamine deprivation inhibits pancreatic cancer growth (A,B) The proliferation of SW1990 (A) and SU.8686 (B) cells cultured in 0 mM glutamine, 2 mM glutamine, or 2 mM glutamine plus Don was compared using Cell Counting Kit-8. (C‒F) Tumor growth (C), tumor volume (D), tumor weight (E), and harvested tumors (F) in BALB/c nude mice injected with SW1990 cells treated with or without Don (750 μg/kg) were measured at 30 days after tumor inoculation. (G‒I) Tumor growth (G), tumor volume (H), and tumor weight (I) in BALB/c nude mice injected with BxPC-3 cells treated with or without Don (750 μg/kg) were measured at 30 days after tumor inoculation. ns, not significant; ** P<0.01, *** P<0.001, **** P<0.0001.

The avidity of glutamine for cancer cells has been well recognized because glutamine is a common component of cell culture media. To examine the effect of glutamine on pancreatic cancer growth, the proliferation of pancreatic cancer cells (SW1990 and SU.8686) at different time points was determined by CCK-8. Glutamine enhanced the proliferation of pancreatic cancer cells, which was inhibited by Don ( Figure 1A,B). In addition, glutamine blockade using Don inhibited tumor volume and weight in SW1990 and BxPC-3 mouse xenograft models ( Figure 1C‒I). Therefore, glutamine is an essential nutrient for the progression of pancreatic cancer.

### Glutamine starvation induces ferroptosis in pancreatic cancer cells

**Figure 2:**
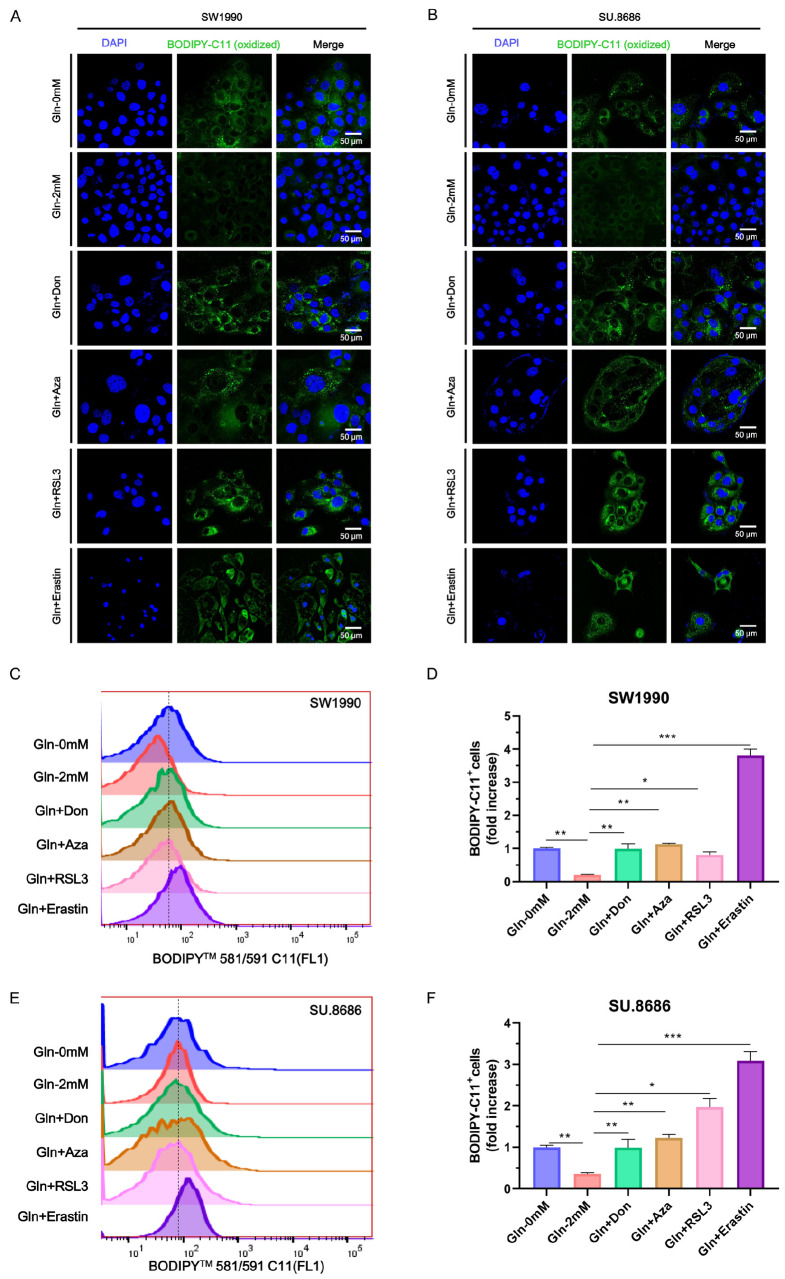
Figure 2 Deprivation of glutamine induces ferroptosis in pancreatic cancer cells (A,B) Fluorescence images of BODIPY-C11-stained SW1990 (A) and SU.8686 (B) pancreatic cancer cells cultured for 48 h in glutamine-free medium, medium containing 2 mM glutamine, or medium containing 2 mM glutamine supplemented with 50 μM diazooxonorleucine (Don) or 10 μM azaserine (Aza). Cells treated with 10 μM RSL3 or 20 μM erastin were used as positive controls. Blue, DAPI; green, BODIPY-C11 (oxidized). (C‒F) Proportion of BODIPY-C11 evaluated by flow cytometry in SW1990 (C,D) and SU.8686 (E,F) cells cultured for 48 h in glutamine-free medium, medium containing 2 mM glutamine, or media containing 2 mM glutamine supplemented with 50 μM Don or 10 μM Aza. ns, not significant; * P<0.05, ** P<0.01, *** P<0.001.

**Figure 3:**
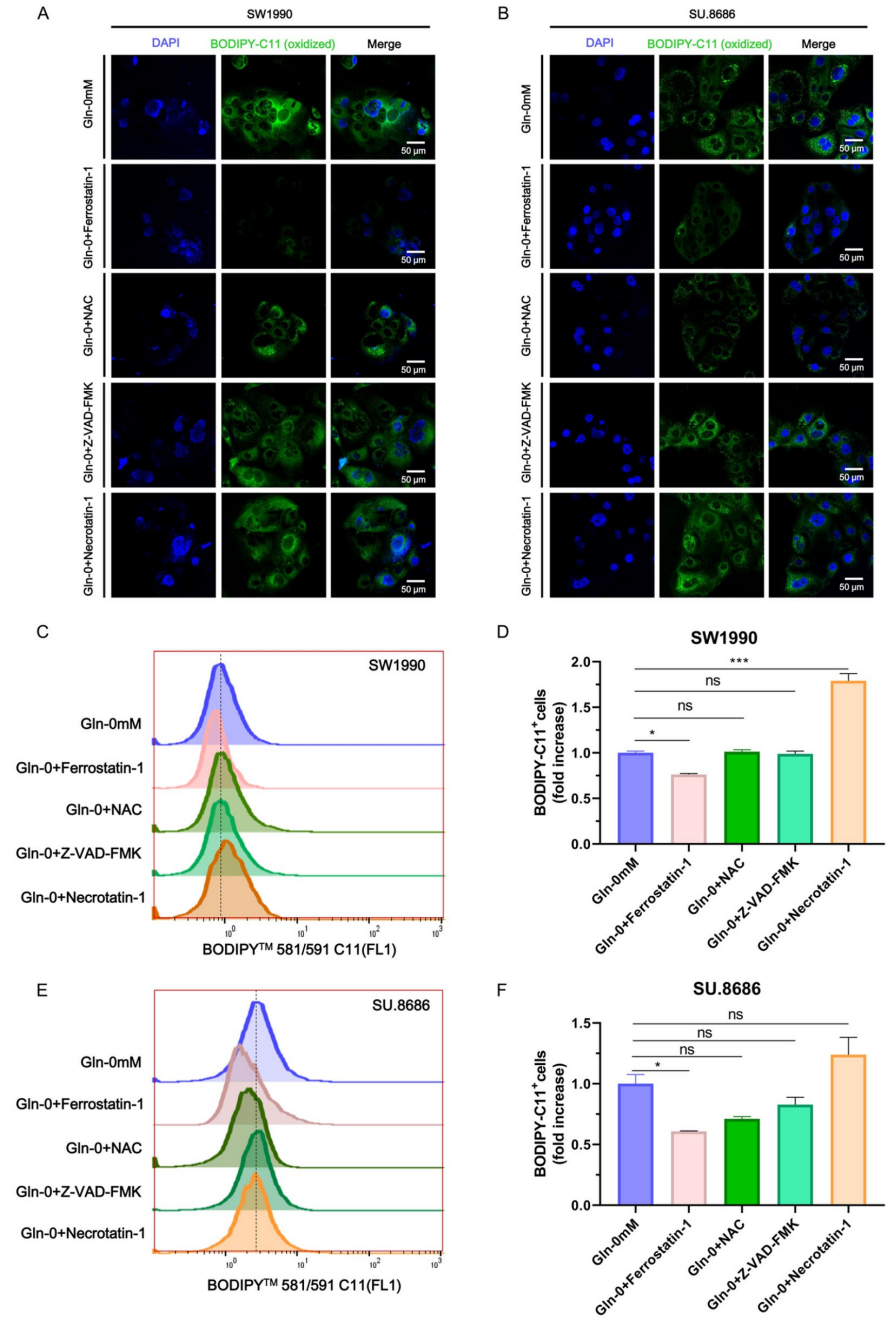
Figure 3 Glutamine-induced ferroptosis could be rescued by ferrostatin (A,B) Fluorescence images of BODIPY-C11-stained SW1990 (A) and SU.8686 (B) pancreatic cancer cells cultured in glutamine-free medium alone or glutamine-free medium supplemented with 2 μM ferrostatin-1, 5 mM N-acetylcysteine, 50 μM Z-VAD-FMK, or 150 μM necrostatin-1 for 48 h. Blue, DAPI; green, BODIPY-C11 (oxidized). (C‒F) Proportion of BODIPY-C11 evaluated by flow cytometry in SW1990 (C,D) and SU.8686 (E,F) cells. ns, not significant; * P<0.05; *** P<0.001.

BODIPY-C11 is a lipid peroxidation sensor used to detect ROS in cells and membranes, with good spectral separation of the nonoxidized (590 nm) and oxidized (510 nm) forms [17]. To investigate the effect of glutamine starvation on ferroptosis in pancreatic cancer cells, BODIPY-C11+ cells were assessed by immunofluorescence assay and flow cytometry. Ferroptosis-inducing agents (RSL3 and erastin) were used as positive controls. The levels of ferroptosis in SW1990 and SU.8686 cells deprived of glutamine were higher than those in cells supplemented with 2 mM glutamine as revealed by confocal laser scanning microscopy ( Figure 2A,B). Additionally, glutamine inhibitors (Don and Aza) induced ferroptosis in pancreatic cancer cells ( Figure 2A,B). The same trend of glutamine-induced ferroptosis in pancreatic cancer cells was confirmed by flow cytometry ( Figure 2C‒F). A major proportion of ferroptotic cells were lost during the washing process of the immunofluorescence assay, and ferroptosis was more evident in the erastin group. This led to an inconsistency between the Erastin and RSL3 controls that underwent immunofluorescence and flow cytometry assays. The inhibitors of apoptosis (Z-VAD-FMK) and necroptosis (necrostatin-1) failed to block glutamine starvation-induced lipid oxidation in SW1990 ( Figure 3A,C,D) and SU.8686 cells ( Figure 3B,E,F). In contrast, the ferroptosis inhibitor ferrostatin-1 efficiently blocked cell lipid oxidation in response to glutamine starvation ( Figure 3C‒F). The ferroptosis inhibitor NAC efficiently blocked lipid oxidation in SU.8686 cells, but not in SW1990 cells, in response to glutamine starvation. Therefore, glutamine deprivation induces ferroptosis in pancreatic cancer cells *in vitro*.

### Deprivation of glutamine induces ferroptosis in pancreatic cancer *in vivo*

**Figure 4:**
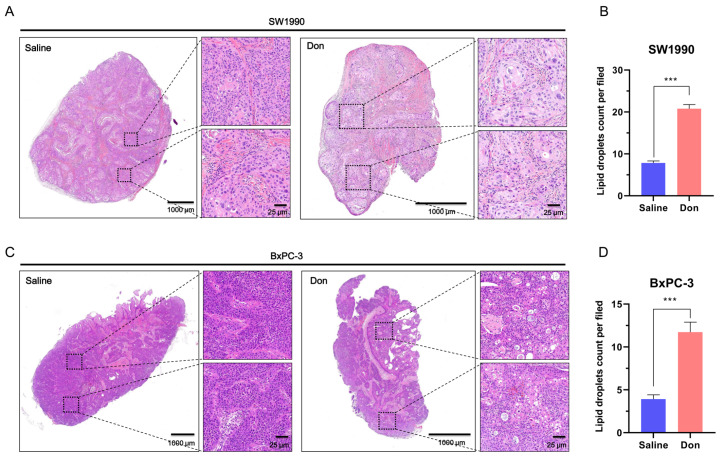
Figure 4 Deprivation of glutamine induces pancreatic cancer cell ferroptosis *in vivo* (A‒D) Hematoxylin and eosin (H&E)-stained sections of tumor tissue from BALB/c nude mice injected with SW1990 (A,B) and BxPC-3 (C,D) pancreatic cancer cells treated with saline (left) or diazooxonorleucine (Don) (right). *** P<0.001.

Lipid droplet accumulation is a feature of ferroptosis in cancer cells and can be directly observed by histopathological examination. The accumulation of lipid droplets is related to the induction of ferroptosis [ 18, 19] . In clear cell renal cell carcinoma, acyl-CoA synthetase 3 (ACSL3) could regulate ferroptosis susceptibility in a manner dependent on lipid droplet accumulation by metabolizing exogenously derived lipids [19]. Inhibition of ACSL3 reduces the sensitivity of cancer cells to ferroptotic cell death. To examine glutamine inhibition-induced ferroptosis *in vivo*, xenograft mouse models, developed by injecting pancreatic cancer cells, were treated with saline or Don. Athymic BALB/c nude mice were subcutaneously inoculated with SW1990 or BxPC-3 cells. Hematoxylin and eosin (H&E) staining revealed increased lipid peroxide droplet accumulation in the Don group compared to the control group ( Figure 4), indicating that deprivation of glutamine induces ferroptosis in pancreatic cancer cells *in vivo*.

### Glutamine decreases ROS production in pancreatic cancer cells

**Figure 5:**
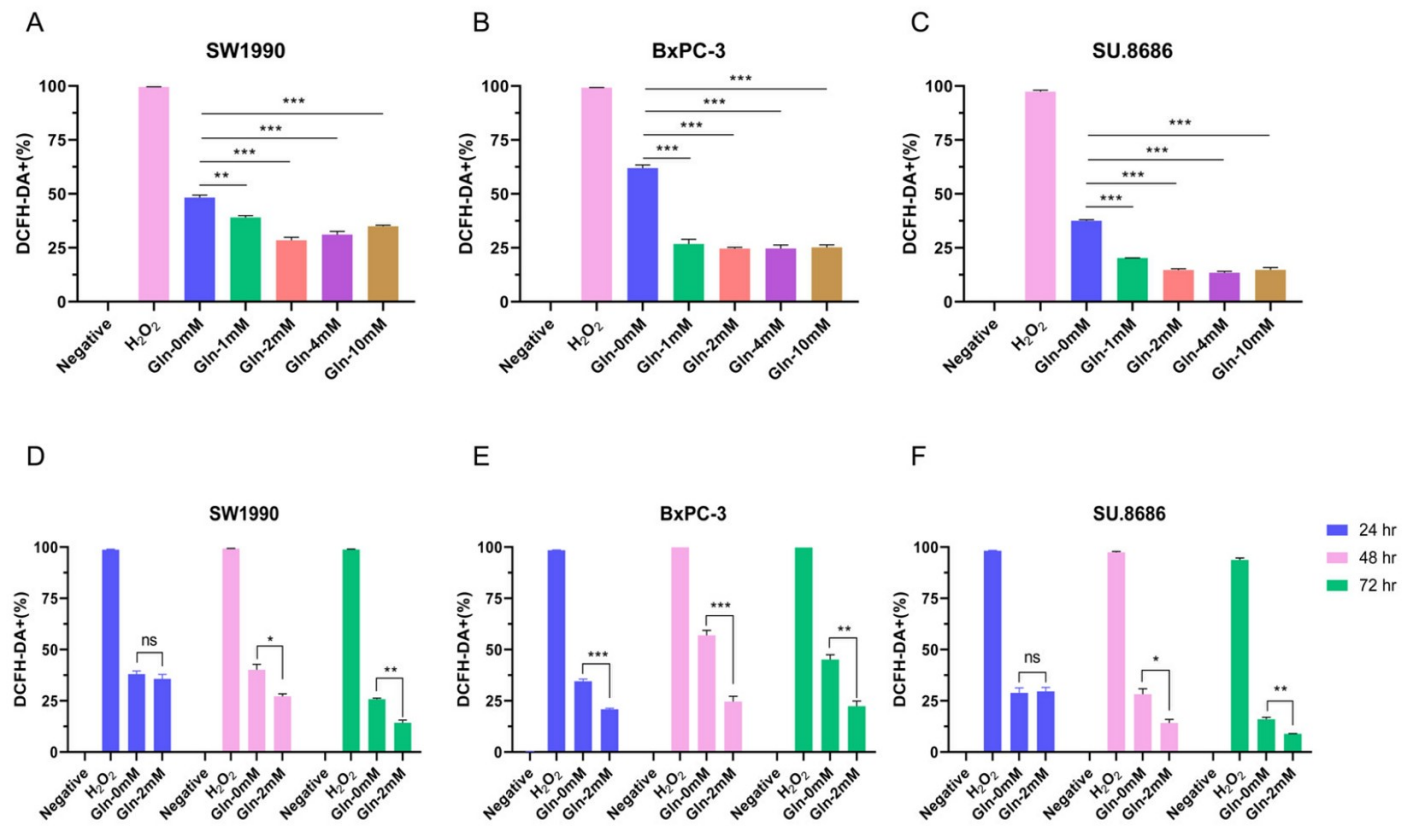
Figure 5 Glutamine blockade promotes reactive oxygen species (ROS) formation in pancreatic cancer cells (A‒C) The proportion of 2’,7’-dichlorodihydrofluorescein diacetate-positive cells (DCFH-DA +) was evaluated by flow cytometry in SW1990, BxPC-3 and SU.8686 pancreatic cancer cells that were cultured in glutamine-free medium or medium with different concentrations of glutamine (1 mM,2 mM,4 mM, and 10 mM) for 72 h. (D‒F) The proportion of DCFH-DA + cells was examined by flow cytometry in SW1990, BxPC-3, and SU.8686 cells cultured for 24, 48, and 72 h, in glutamine-free medium or medium containing 2 mM glutamine. Cells treated with 100 μM H 2O 2 were used as positive controls. ns, not significant; * P<0.05, ** P<0.01; *** P<0.001.

Previous studies have indicated that glutamine deficiency increases ROS levels in cancer cells [18]. DCFH-DA is one of the most commonly used probes for quantifying cellular H _2_O _2_ and ROS [19]. To evaluate the effect of glutamine deficiency on ROS production during pancreatic cancer, SW1990, BxPC-3, and SU.8686 cells were cultured in media with varying concentrations of glutamine (0, 1, 2, 4, and 10 mM) and examined by DCFH-DA assay using flow cytometry. Since commercially available media usually contain 2 mM glutamine, cells were cultured for different durations (24, 48, and 72 h) in media containing 2 mM glutamine. Glutamine inhibited ROS formation in pancreatic cancer cells in a concentration-dependent manner ( Figure 5A‒C). The decrease in ROS was more evident when the cells were cultured for 48 or 72 h ( Figure 5D‒F). BxPC-3 cells were more dependent on glutamine than SW1990 and SU.8686 cells. Thus, glutamine decreases ROS production in pancreatic cancer cells.

### Glutamine decreases ROS formation via reduced glutathione (GSH) production in pancreatic cancer cells

**Figure 6:**
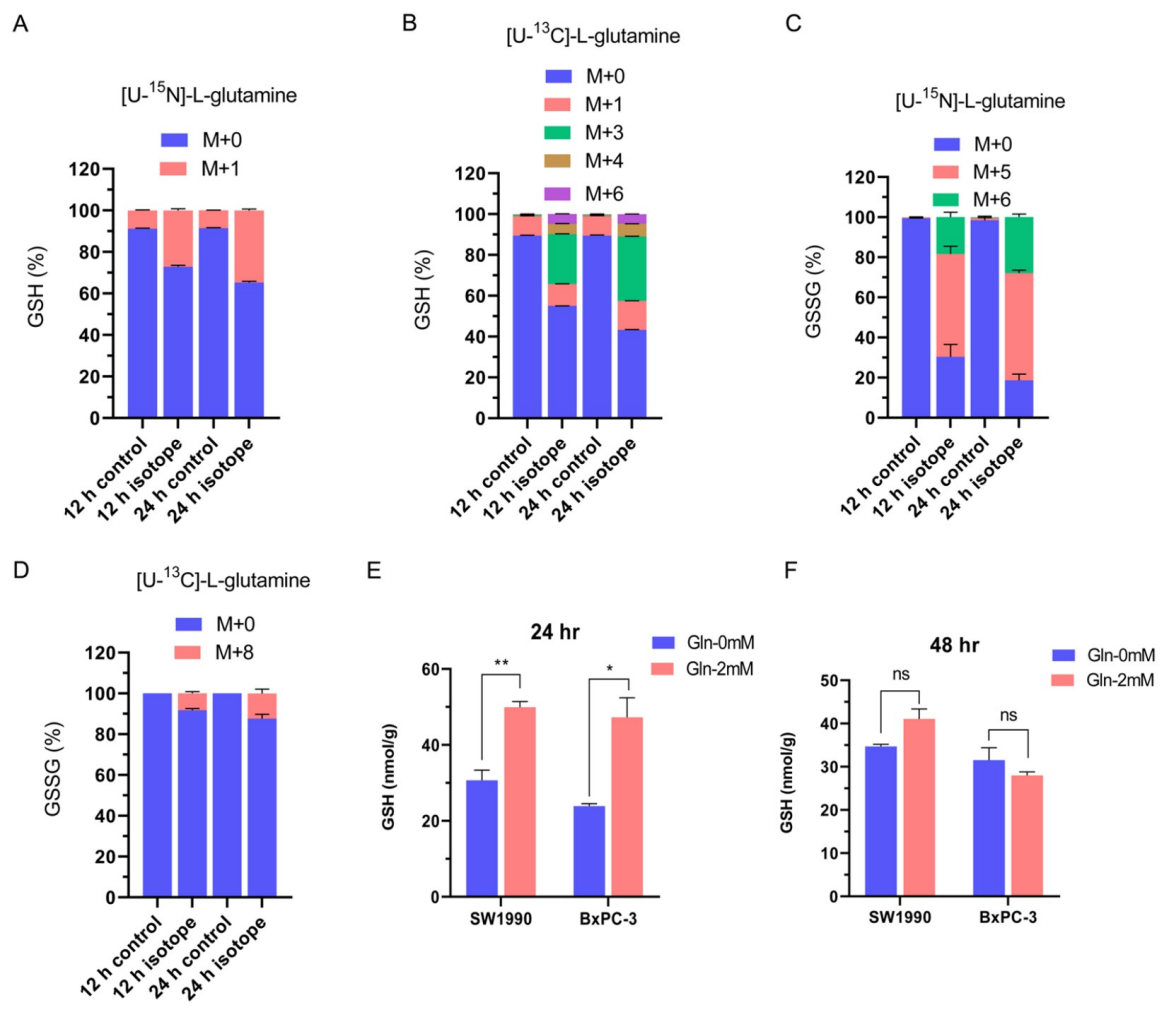
Figure 6 Glutamine promotes reduced glutathione (GSH) production in pancreatic cancer cells (A‒D) Fractional isotopomer labelling of GSH (A,B) and oxidized glutathione (GSSG) (C,D) measured by culturing SU.8686 cells with [U- 13C]-L-glutamine or [U- 15N]-L-glutamine for 12 and 24 h was assessed using ultrahigh-performance liquid chromatography (UHPLC)-QE-MS. M+ (M0, M1 to Mn) represents the number of C or N in metabolites labelled by isotopes. The fractional abundance of mass isotopomers defined by M0, M1, and Mn was calculated. (E,F) Alteration of GSH in SW1990 and BxPC-3 pancreatic cancer cells cultured in media with or without 2 mM glutamine for 24 h (E) or 48 h (F) was detected by UHPLC-MS/MS. ns, not significant; * P<0.05; ** P<0.01.

Glutathione (GSH) aids in the neutralization of free radicals and is important for protecting cells against oxidative stress and toxic xenobiotics. The effect of glutamine on ROS production may be associated with GSH and oxidized glutathione (GSSG) production. To test this hypothesis, fractional isotopomer labelling of GSH and GSSG with [U- ^13^C]-L-glutamine or [U- ^15^N]-L-glutamine was examined by UHPLC in SU.8686 cells cultured for 12 and 24 h. Next, isotope-labelled glutamine was traced in the GSH and GSSG produced ( Figure 6A‒D). The concentration of GSH, measured by UHPLC-MS/MS, was higher in SW1990 and BxPC-3 cells treated with medium containing 2 mM glutamine than in cells cultured in glutamine-free media for 24 h but not for 48 h ( Figure 6E,F). Glutamine supplementation was not associated with GSSG production in cells cultured for 24 h or 48 h (data not shown). The deprivation of glutamine led to a decrease in intracellular GSH at 24 h and an increase in intracellular ROS at 48 h. This could be explained by the production of GSH being decreased at first and the ROS levels subsequently increased [20]. Thus, glutamine blockade promotes ROS formation by reducing GSH but not GSSG in pancreatic cancer cells.

### Glutamine blockade promotes ROS formation in pancreatic cancer cells

**Figure 7:**
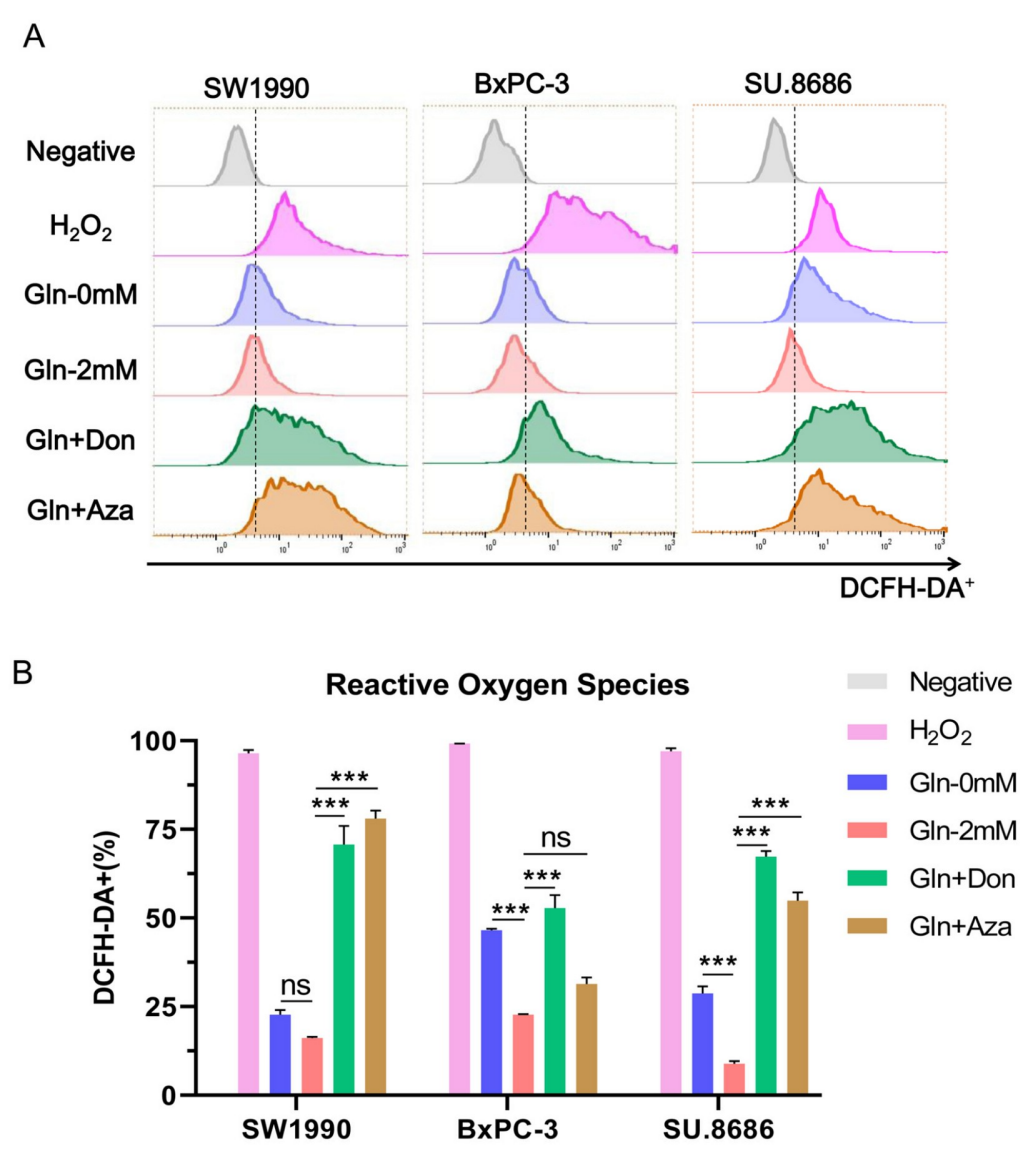
Figure 7 Glutamine blockade promotes reactive oxygen species (ROS) formation in pancreatic cancer cells (A,B) Representative histogram and proportion of 2’,7’-dichlorodihydrofluorescein diacetate-positive (DCFH-DA +) cells were evaluated by flow cytometry in SW1990, BxPC-3 and SU.8686 cells cultured for 72 h in glutamine-free medium, medium containing 2 mM glutamine, or media containing 2 mM glutamine supplemented with 50 μM diazooxonorleucine (Don) or 10 μM azaserine (Aza). Cells treated with H 2O 2(100 μM) were used as positive controls. ns, not significant; *** P<0.001.

Our results have shown that glutamine decreases ferroptosis in pancreatic cancer, which can be reversed by glutamine inhibitors such as Don and Aza. To confirm that these inhibitors induce ferroptosis by promoting ROS levels, the proportion of DCFH-DA was evaluated in SW1990, BxPC-3, and SU.8686 cells cultured in media containing 2 mM glutamine and treated with Don or Aza for 72 h. Glutamine blockade promoted ROS formation in pancreatic cancer cells (SW1990 and SU.8686), as measured by flow cytometry ( Figure 7).

### Potential molecular pathways involved in glutamine deprivation-induced ferroptosis

**Figure 8:**
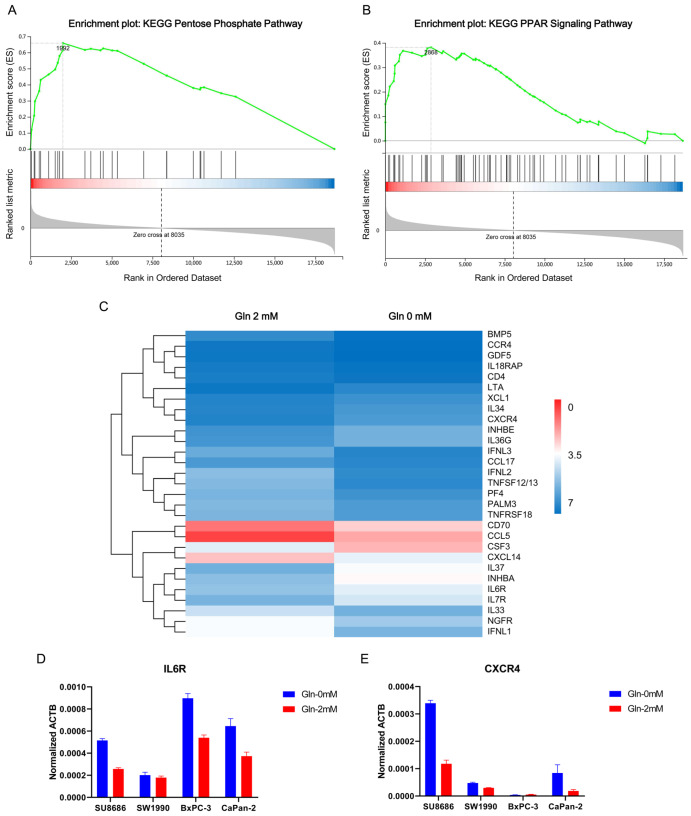
Figure 8 Gene expression profiles of pancreatic cancer cells treated with or without 2 mM glutamine examined by RNA-seq (A, B) Signaling pathways, including the pentose phosphate pathway (A) and peroxisome proliferator-activated receptor signaling pathway (B), were enriched by gene set enrichment analysis (GSEA). (C) The cytokine‒cytokine receptor interaction pathway was enriched in cells cultured in medium containing 2 mM glutamine. (D,E) The differential expression of IL6R and CXCR4 was confirmed by quantitative RT-PCR.

To elucidate the potential molecular alterations involved in glutamine deprivation, gene expression profiles of pancreatic cancer cells (SW1990, BxPC-3, SU.8686, CAPAN-1, CAPAN-2, CFPAC-1, Panc-1, and MiaPaCa-2) treated with or without 2 mM glutamine were examined by RNA-seq. Data were analyzed using KEGG and GSEA computational tools. The pentose phosphate pathway (PPP), a metabolic pathway parallel to glycolysis that produces precursors for nucleotide synthesis, was enriched by GSEA, suggesting that glutamine supplies substrates for pancreatic cancer growth ( Figure 8A). Peroxisome proliferator-activated receptors (PPARs), which play essential roles in the regulation of cellular differentiation, development, metabolism (carbohydrates, lipids, and proteins), and tumorigenesis, were also enriched ( Figure 8B).

Tumor cells can reprogram their metabolism under nutrient-deprived conditions. In this study, the cytokine‒cytokine receptor interaction pathway was the most enriched pathway between pancreatic cancer cells treated with and without glutamine, including *CCL5*, *CCR4*, *LTA*, *CXCR4*, *IL-6R*, and *IL-7R* ( Figure 8C). The differential expression of *IL6R* and *CXCR4* was confirmed by qRT-PCR ( Figure 8D,E). Therefore, pancreatic cancer cells may secrete cytokines under glutamine deprivation conditions to recruit stromal or immune cells to cope with nutrient crises.

## Discussion

**Figure 9:**
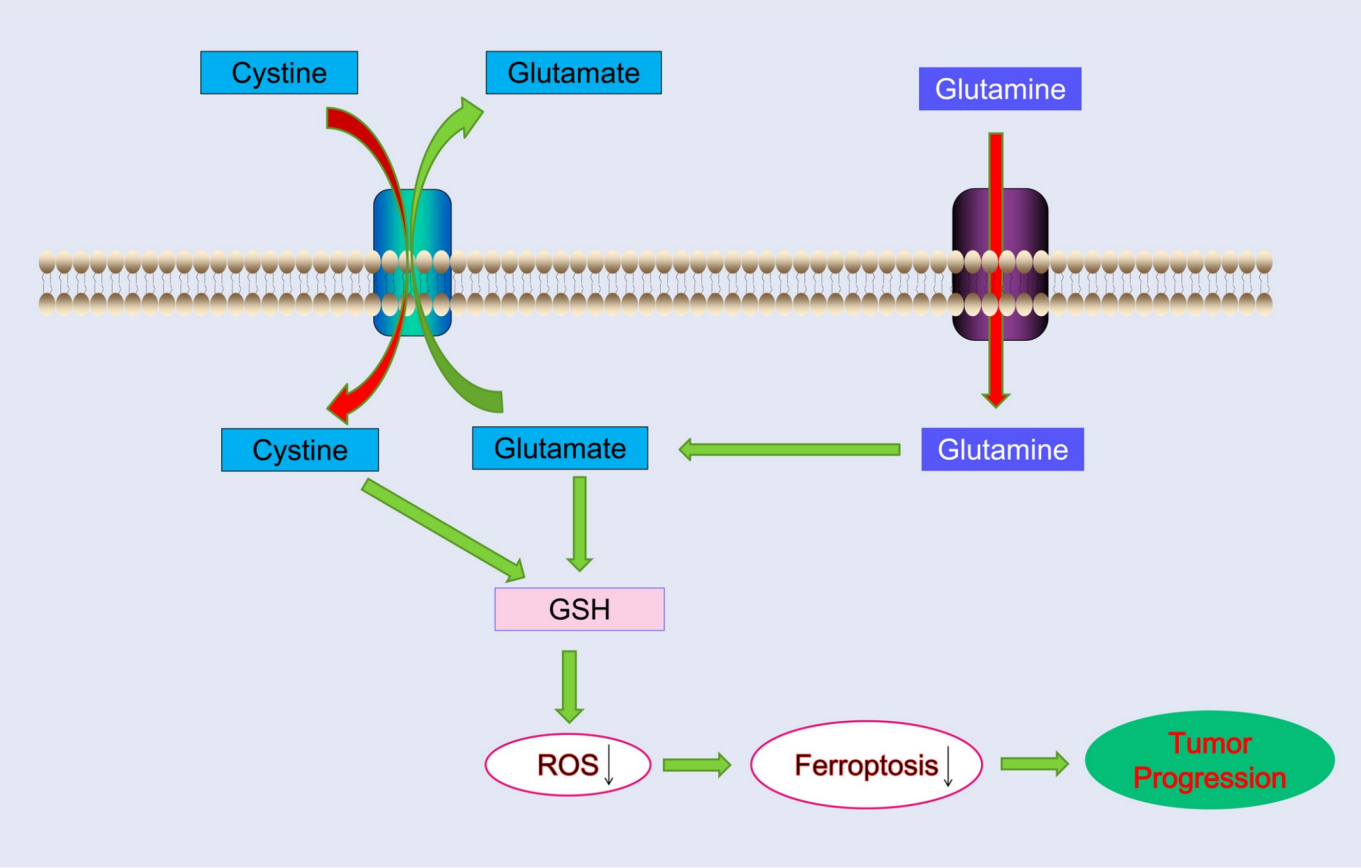
Figure 9 Schematic illustration of how glutamine promotes pancreatic cancer progression by inhibiting reactive oxygen species (ROS)-related ferroptosis Glutamine deprivation reduces GSH production and promotes ROS production, which may trigger ferroptosis in pancreatic cancer cells.

Glutamine is a super nutrient for pancreatic cancer growth and progression [ 10, 12] . In this study, we demonstrated that glutamine deprivation inhibits pancreatic cancer growth by inducing ferroptosis, which may be associated with ROS production. Glutamine inhibitors increase ferroptosis in pancreatic cancer cells. Induction of ferroptosis upon glutamine deprivation can be rescued by a ferroptosis inhibitor (ferrostatin-1) but not by apoptosis inhibitor (Z-VAD-FMK) or necroptosis inhibitor (necrostatin-1). Signaling pathways, including PPP, PPARs, and cytokine‒cytokine receptor interactions, were enriched in glutamine-starved cells. Therefore, pancreatic cancer cells require exogenous glutamine to prevent ferroptosis. Glutamine deprivation reduces GSH production and promotes ROS production, which may induce ferroptosis in pancreatic cancer cells ( Figure 9). However, further studies are needed to explore the association between glutamine deprivation and ferroptosis in pancreatic cancer cells.

Ferroptosis has been demonstrated to be associated with metabolism, redox biology, and cancer [ 4, 5] . Triggering ferroptosis is a potential strategy for cancer treatment [ 4, 21] . Previous reports have suggested a potential connection between glutamine and ferroptosis [18]. Glutamine-fueled intracellular metabolic pathways play an essential role in ferroptosis through the product of glutaminolysis, α-ketoglutarate (α-KG) [18]. Accumulation of extracellular glutamate, an interacting form of glutamine, can stimulate ferroptosis in physiological contexts [22]. Our study confirms that glutamine deprivation activates ferroptosis in pancreatic cancer cells.

Glutamine deprivation is closely related to ROS production, which induces ferroptosis in tumors [ 11, 22, 23] . ROS levels are dynamically modulated throughout pancreatic cancer progression, with lower levels of ROS accelerating tumor initiation in premalignant conditions and higher levels promoting metastatic progression [24]. Although ROS is pro-tumorigenic, high ROS levels are cytotoxic to cancer cells [22]. Tumor cells express increased levels of antioxidant proteins to detoxify ROS, suggesting that a delicate balance in intracellular ROS levels is required for optimal cancer cell function. Upon the modulation of oncogenic *KRAS*, glutamine is converted into oxaloacetate by aspartate transaminase and further into pyruvate, which increases the NADPH/NADP ^+^ ratio to preserve cellular redox balance [11]. Our study confirmed a previous report showing that glutamine deprivation results in an increase in ROS and a decrease in GSH levels [11]. Meanwhile, the percentage of DCFH-DA ^+^ cells shows a decreasing trend over time in pancreatic cancer cells cultured in glutamine-free media, which may be caused by the adaptation of cells to glutamine deprivation through glutamine anaplerotic reactions [8].

Thus, the association between glutamine deprivation and ROS production may be related to cysteine production. Cysteine is a source of glutathione synthesis that participates in counteracting ROS production through oncogenic signaling. Cyst(e)ine supplementation promotes colon cancer growth and chemoresistance by activating mTORC1 via GCN2-ATF4. This quenches chemotherapy-induced ROS via the synthesis of glutathione [25]. Glutamate, cysteine, and glycine are precursor amino acids involved in GSH synthesis. Inhibition of cytosolic aspartate aminotransaminase (GOT1) enhances pancreatic cancer cell death via ferroptosis. Furthermore, cysteine, glutathione, and lipid antioxidants function as metabolic vulnerabilities following GOT1 blockade [26]. Glutamate is exchanged for cystine in a 1:1 ratio by system Xc ^_^, a plasma membrane transporter. Thus, glutamate or glutamine level may impact ferroptosis through cysteine transport [4]. A previous study showed that cysteine depletion could induce pancreatic cancer ferroptosis in genetically engineered mice and inhibit pancreatic cancer growth by mediating the synthesis of GSH and coenzyme A [21]. The deletion of a system Xc ^_^ subunit, SLC7A11, or a combination of GSH and coenzyme A blockade, or the administration of cyst(e)inases, could induce tumor-selective ferroptosis [21]. Therefore, we suggest that glutamine is an essential nutritional source that not only supports pancreatic cancer growth but also prevents the induction of ferroptosis. However, further studies are needed to elucidate the mechanism of glutamine deprivation-induced cell death.

This study indicates a potential connection between glutamine, cysteine, GSH, ROS, and ferroptosis in pancreatic cancer. However, the potential molecules regulating this metabolic pathway need to be further identified. Nuclear factor erythroid 2-related factor 2 (NRF2) is a basic leucine zipper protein and master regulator of the cellular antioxidant response [27]. It is a transcription factor that regulates the expressions of antioxidant proteins that protect cells against ROS damage triggered by injury and inflammation [27]. ROS levels are tightly regulated by the NRF2/Kelch-like ECH-associated protein 1 (KEAP1) interaction under normal physiological conditions. In pancreatic cancer, mutations in the *KRAS* proto-oncogene are present in over 90% of cases and are associated with tumor proliferation and cellular metabolism. A byproduct of *KRAS* oncogenic activation is the increased production of ROS, which is harmful to cellular components. To compensate for this, the *KRAS* G12D mutation increases the constitutive transcription of *NRF2* to disrupt the NRF2-KEAP1 interaction, thereby actively suppressing intracellular ROS via the Raf-MEK-ERK-Jun pathway [28]. Thus, enhanced ROS detoxification and the antioxidant function of NRF2 may play an unappreciated role in tumorigenesis. KRAS/NRF2 ^-^ and KRAS/KEAP1-mutant lung cancers are sensitive to reduced glutamine levels [7]. Moreover, nutrient deprivation is sufficient to enhance the biosynthesis and/or secretion of specific proinflammatory cytokines to trigger adaptive responses [29]. A previous study showed that glutamine restriction stimulates the release of IL-6 and IL-8, which further recruits B cells, macrophages, and neutrophils. Our study shows that the cytokine‒cytokine receptor interaction pathway is enriched under glutamine deprivation, indicating that pancreatic cancer cells may secrete cytokines to recruit stromal cells to cope with glutamine deprivation. The association between glutamine deprivation, NRF2/KEAP1, and cytokine secretion in KRAS-mutant pancreatic cancer warrants further investigation. The distinctive effects of NAC on ferroptosis may be explained by the differential expression of KRAS (SW1990, KRAS low; SU.8686 KRAS high), which plays a key role in regulating the ROS reaction via the NRF2-KEAP1 interaction [30].

In summary, glutamine has been confirmed to be a critical nutrient for the oncogenesis and progression of pancreatic cancer. Our study further demonstrates that glutamine deprivation can induce pancreatic cancer ferroptosis. This provides necessary evidence to explore the efficacy of glutamine blockade in pancreatic cancer. However, since glutamine is an important nutrient for the human body, the delicate balance between its antitumour effect and potential toxicity remains to be explored. Further studies are required to elucidate the mechanism by which necrostatin-1 induces ferroptosis in pancreatic cancer cells.

## References

[1] Mizrahi JD, Surana R, Valle JW, Shroff RT. Pancreatic cancer.
Lancet 2020, 395: 2008–2020. https://doi.org/10.1016/s0140-6736(20)30974-0.

[2] Oba A, Ho F, Bao QR, Al-Musawi MH, Schulick RD, Del Chiaro M (2020). Neoadjuvant treatment in pancreatic cancer. Front Oncol.

[3] Liu M, Shi Y, Hu Q, Qin Y, Ji S, Liu W, Zhuo Q (2021). SETD8 induces stemness and epithelial-mesenchymal transition of pancreatic cancer cells by regulating ROR1 expression. Acta Biochim Biophys Sin.

[4] Stockwell BR, Friedmann Angeli JP, Bayir H, Bush AI, Conrad M, Dixon SJ, Fulda S (2017). Ferroptosis: a regulated cell death nexus linking metabolism, redox biology, and disease. Cell.

[5] Ye Z, Zhuo Q, Hu Q, Xu X, Mengqi liu X, Zhang Z, Xu W (2021). FBW7-NRA41-SCD1 axis synchronously regulates apoptosis and ferroptosis in pancreatic cancer cells. Redox Biol.

[6] Dolma S, Lessnick SL, Hahn WC, Stockwell BR (2003). Identification of genotype-selective antitumor agents using synthetic lethal chemical screening in engineered human tumor cells. Cancer Cell.

[7] Romero R, Sayin VI, Davidson SM, Bauer MR, Singh SX, LeBoeuf SE, Karakousi TR (2017). Keap1 loss promotes Kras-driven lung cancer and results in dependence on glutaminolysis. Nat Med.

[8] Xu R, Yang J, Ren B, Wang H, Yang G, Chen Y, You L (2020). Reprogramming of amino acid metabolism in pancreatic cancer: recent advances and therapeutic strategies. Front Oncol.

[9] Altman BJ, Stine ZE, Dang CV (2016). From Krebs to clinic: glutamine metabolism to cancer therapy. Nat Rev Cancer.

[10] Bernfeld E, Foster DA (2019). Glutamine as an essential amino acid for KRas-driven cancer cells. Trends Endocrinol Metab.

[11] Son J, Lyssiotis CA, Ying H, Wang X, Hua S, Ligorio M, Perera RM (2013). Glutamine supports pancreatic cancer growth through a KRAS-regulated metabolic pathway. Nature.

[12] Leone RD, Zhao L, Englert JM, Sun IM, Oh MH, Sun IH, Arwood ML (2019). Glutamine blockade induces divergent metabolic programs to overcome tumor immune evasion. Science.

[13] Sauvat A, Wang Y, Segura F, Spaggiari S, Müller K, Zhou H, Galluzzi L (2015). Quantification of cellular viability by automated microscopy and flow cytometry. Oncotarget.

[14] Hong X, Qiao S, Li F, Wang W, Jiang R, Wu H, Chen H (2020). Whole-genome sequencing reveals distinct genetic bases for insulinomas and non-functional pancreatic neuroendocrine tumours: leading to a new classification system. Gut.

[15] Han K, Hua J, Zhang Q, Gao Y, Liu X, Cao J, Huo N (2021). Multi-residue analysis of fipronil and its metabolites in eggs by SinChERS-based UHPLC-MS/MS. Food Sci Anim Resour.

[16] Smith CA, Want EJ, O′Maille G, Abagyan R, Siuzdak G (2006). XCMS:  processing mass spectrometry data for metabolite profiling using nonlinear peak alignment, matching, and identification. Anal Chem.

[17] Zou Y, Palte MJ, Deik AA, Li H, Eaton JK, Wang W, Tseng YY (2019). A GPX4-dependent cancer cell state underlies the clear-cell morphology and confers sensitivity to ferroptosis. Nat Commun.

[18] Gao M, Monian P, Quadri N, Ramasamy R, Jiang X (2015). Glutaminolysis and transferrin regulate ferroptosis. Mol Cell.

[19] Wu D, Yotnda P (2011). Production and detection of reactive oxygen species (ROS) in cancers. J Vis Exp.

[20] Liu T, Sun L, Zhang Y, Wang Y, Zheng J (2022). Imbalanced GSH/ROS and sequential cell death. J Biochem Mol Tox.

[21] Badgley MA, Kremer DM, Maurer HC, DelGiorno KE, Lee HJ, Purohit V, Sagalovskiy IR (2020). Cysteine depletion induces pancreatic tumor ferroptosis in mice. Science.

[22] Hayes JD, Dinkova-Kostova AT, Tew KD (2020). Oxidative stress in cancer. Cancer Cell.

[23] Durand N, Storz P (2017). Targeting reactive oxygen species in development and progression of pancreatic cancer. Expert Rev Anticancer Ther.

[24] Cheung EC, DeNicola GM, Nixon C, Blyth K, Labuschagne CF, Tuveson DA, Vousden KH (2020). Dynamic ROS control by TIGAR regulates the initiation and progression of pancreatic cancer. Cancer Cell.

[25] Wu J, Yeung SCJ, Liu S, Qdaisat A, Jiang D, Liu W, Cheng Z (2021). Cyst(e)ine in nutrition formulation promotes colon cancer growth and chemoresistance by activating mTORC1 and scavenging ROS. Sig Transduct Target Ther.

[26] Kremer DM, Nelson BS, Lin L, Yarosz EL, Halbrook CJ, Kerk SA, Sajjakulnukit P (2021). GOT1 inhibition promotes pancreatic cancer cell death by ferroptosis. Nat Commun.

[27] Rojo de la Vega M, Chapman E, Zhang DD (2018). NRF2 and the hallmarks of cancer. Cancer Cell.

[28] DeNicola GM, Karreth FA, Humpton TJ, Gopinathan A, Wei C, Frese K, Mangal D (2011). Oncogene-induced Nrf2 transcription promotes ROS detoxification and tumorigenesis. Nature.

[29] Shanware NP, Bray K, Eng CH, Wang F, Follettie M, Myers J, Fantin VR (2014). Glutamine deprivation stimulates mTOR-JNK-dependent chemokine secretion. Nat Commun.

[30] Gorrini C, Harris IS, Mak TW (2013). Modulation of oxidative stress as an anticancer strategy. Nat Rev Drug Discov.

